# Animal TSEs and public health: What remains of past lessons?

**DOI:** 10.1371/journal.ppat.1006759

**Published:** 2018-02-08

**Authors:** Saima Zafar, Mohsin Shafiq, Olivier Andréoletti, Inga Zerr

**Affiliations:** 1 Clinical Dementia Center and DZNE, Neurology, Georg-August University, University Medical Center Göttingen (UMG), Göttingen, Germany; 2 UMR INRA ENVT 1225, Interactions Hôtes Agents Pathogènes, Ecole Nationale Vétérinaire de Toulouse, Toulouse, France; Washington University School of Medicine, UNITED STATES

Transmissible spongiform encephalopathies (TSEs) or prion diseases of animals include scrapie in sheep and goat, chronic wasting disease (CWD) in cervids (including mule deer, white-tailed deer, moose, elk, and reindeer), and bovine spongiform encephalopathy (BSE) in cattle; these diseases are either classical/naturally occurring or atypical forms and are thought to be caused by the spontaneous misfolding of prion protein. Marked heterogeneity with respect to clinicopathological features, susceptibility, and infectivity is known across and within the species, but the molecular basis for that phenomenon is still not completely understood, except for some factors. In most species, susceptibility to the disease is strongly influenced by the polymorphism in prion protein encoding gene (PRNP). In humans, the dimorphism at *PRNP* codon 129 (substitution of valine for methionine) impacts both the susceptibility and the phenotypes of TSEs [1]. However, sheep with Q171R genotype show maximum resistance, and A136V genotype animals show maximum susceptibility to develop scrapie [2], and a single amino acid modification alters TSEs cross-species transmissions [3].

Concerns and speculation about cross-species transmission of TSEs—particularly transmission of animal TSEs to humans—have existed since the infectious nature of these diseases was demonstrated, but there was no evidence of zoonotic transmission until the link between BSE and a novel human TSE (variant CJD or vCJD) was established in the late 1990s. While the number of vCJD clinical cases identified so far remains low, several studies suggested that 1 out of 2,000 individuals could be vCJD infected but asymptomatic in the United Kingdom population [4].

In Europe, the emergence of vCJD triggered a major sanitary crisis that resulted in the implementation of a strong and coherent policy (EU regulation 999/2001) that aimed at eradicating animal TSEs. As a consequence of this policy, the BSE epidemic has nearly faded, and the EU authorities started to dismantle this policy. This change in the EU TSE doctrine raises questions about the necessity and the means to effectively protect human consumers from exposure to animals’ prions.

## Cattle BSEs

**Fig 1:**
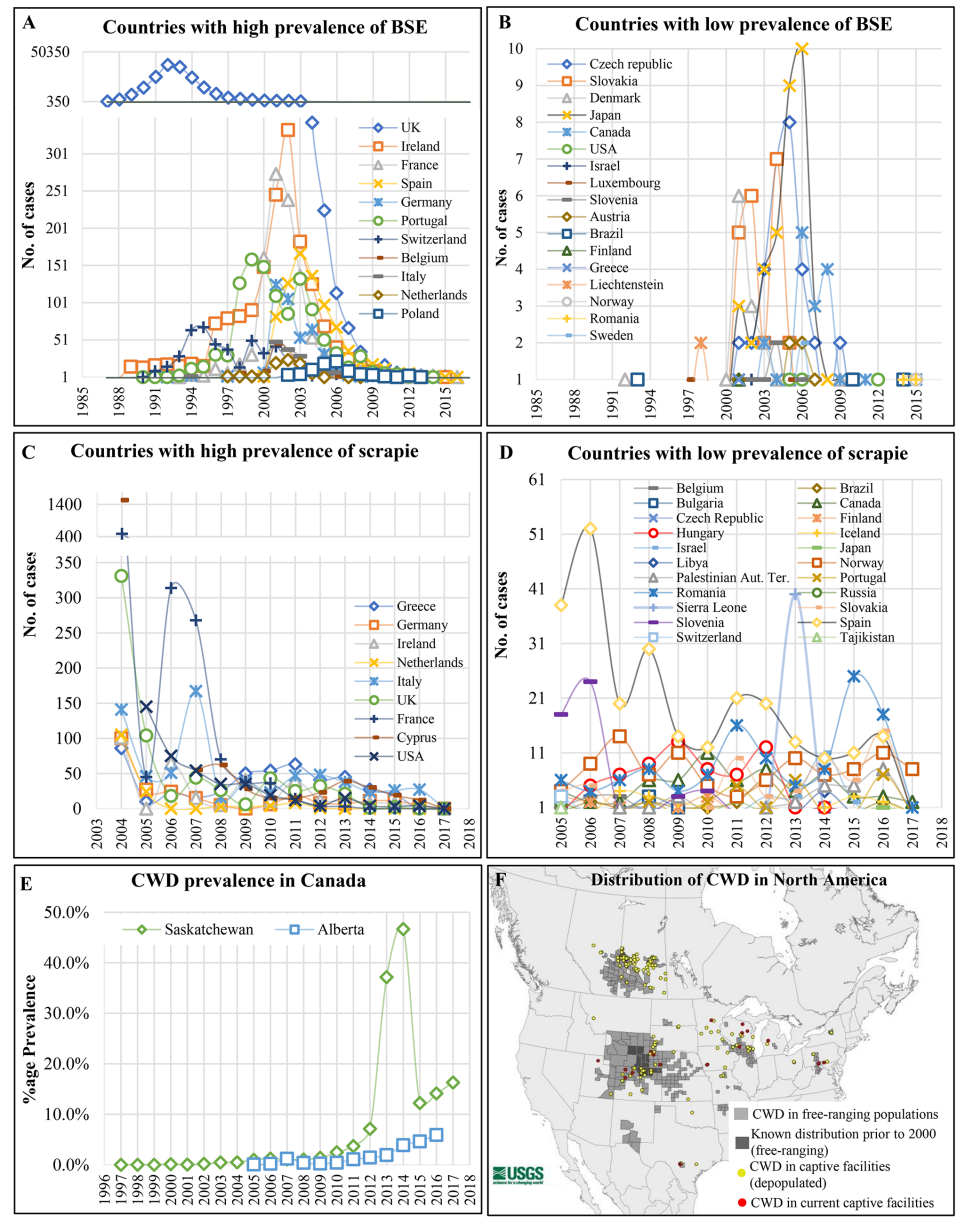
Worldwide occurrence of animal TSEs. (A&B) Show the worldwide prevalence of BSE cases, from 1987 to 2017. Total number of cases shown in C-F is from active and passive surveillance according to the World Organization for animal health, Paris (OIE). (C&D) Show the prevalence of scrapie cases worldwide over the last years, whereas (E) graphs show the percentage of the CWD positive cases of total animals examined for surveillance in the states of Alberta and Saskatchewan, according to the Department of agriculture and forestry, Alberta, and the Canadian Wildlife Health Cooperative. (F) Distribution of CWD in over 20 states of the USA and two Canadian provinces, as of October 2017, according to Geological Survey by the Department of the Interior/USGS, US Geological Survey. CWD, Chronic Wasting Disease.

Classical BSE (C-BSE) was first recognized in 1984–85 as novel TSE affecting cattle in the UK [5]. The epidemics caused about 180,000 clinical cases, and it was estimated that about 400,000 infected animals might have entered the food chain. BSE cases have been identified in Europe and in many other countries including United States of America, Canada, and Japan (Fig 1A & 1B). The National CJD Surveillance Unit UK has estimated that over 1 million infected cattle entered the human food chain [6].

The origin of the BSEs has still not been clearly established, but the number of cases was amplified by the recycling of animal carcasses into cattle feed [7]. The overwhelming weight of evidence from epidemiological and experimental studies supports the view that human dietary exposure to C-BSE caused the emergence of a new TSE in the human-named variant CJD.

The existence of a substantial transmission barrier for BSE to humans probably explains the relatively small size of the vCJD epidemic; but it is likely that the systematic retrieval of tissues that might contain a significant amount of BSE infectivity from the food chain (known as the Specified Risk Material [SRM] policy), which was enforced since 1996 in the UK and 2001 in the rest of the EU, was decisive for preventing the occurrence of new human contaminations.

The implementation, in 2001, of an active surveillance system in EU’s ruminant population, involving mandatory testing of healthy slaughtered cattle and fallen stock in the EU, allowed the discovery of “atypical” BSE. Two major atypical phenotypes were observed, categorized on the basis of the low or high apparent molecular masses of the unglycosylated PrP (resistance form) band, and were termed as bovine amyloidotic spongiform encephalopathy (BASE) or L-BSE [8] and H-BSE [9], respectively. The L-BSE and H-BSE are detected in aged asymptomatic cattle and differ from C-BSE because of the biochemical and transmission properties of PrP^TSE^. The apparent prevalence of atypical BSE cases is low (1.9 cases H-BSE and 1.7 cases L-BSE per million cattle over 8 years old tested) [10]. However, atypical BSE cases have been identified in a number of countries, including in classical BSE-free areas, and the origin of these cases (spontaneous versus acquired) is unknown. In addition, transmission studies in non-human primates and humanized transgenic mice suggest that L-BSE [11] may have the zoonotic potential equal to or greater than C-BSE.

These elements strongly support the view that, while atypical BSE prevalence is currently low, the risk it presents for the human should not be neglected.

## Small ruminants TSE

Classical scrapie in small ruminants is a disease which has been described for several centuries. It was reported for the first time in sheep in the United Kingdom in 1732 and a few years later, in 1759, in Germany. In the following centuries, scrapie endemically affected flocks in many countries [12]. Numerous studies established that classical scrapie can be caused by a variety of Prion strains that can be distinguished by their transmission features in animal models.

In 1998, the spectrum of TSEs in sheep was extended by the discovery in Norway, of an experimentally transmissible, PrP-related neurological disease of sheep (Nor98) that was clearly distinguishable from previously reported scrapie cases (named classical scrapie) and was therefore considered to be an “atypical” form of scrapie [13].

After 2001 and the implementation of active TSE surveillance plans, several similar cases were identified in most EU members states as well in many other countries, including the United States of America and Canada [13]. Atypical scrapie was also detected in Australia and New Zealand [14, 15]—countries that were previously considered as animal TSE-free countries by the OIE (Organisation Mondiale de la Santé Animale, World Organisation for Animal Health) (Fig 1C& 1D).

A retrospective study carried out in tissue banks identified atypical/Nor98 cases in sheep samples collected in the UK in 1987 [16] suggesting that atypical/Nor98 might have been present but undetected in the small ruminant population for decades.

A major concern in scrapie-infected small ruminants (i.e., goats and sheep) is the large distribution of prion infectivity across the tissues, which are in contrast to C-BSE in cattle. The SRM measures (i.e., [i] animal protein feed prohibition to animals, [ii] age-dependent post mortem monitoring system of healthy and at-risk animals [iii] destruction of SRM, and [iv] eradication of herds with confirmed BSE cases) that were implemented in 2001 in the EU, reduced substantially the number of scrapie animals entering the food chain. However, its final efficacy depends on the age (eradication of all animals >30 months of age eradicated from the food chain), the PRNP gene polymorphism of the considered animals, and the prion strain [1, 3].

The zoonotic potential of small ruminant TSE strains remains incompletely characterized. However, results obtained recently in experimental models, i.e., primates (transmission of scrapie prions after a 10-years silent incubation period to cynomolgus macaque) [17] and humanized PrP transgenic mice [11] demonstrated that at least some of these agents have the ability to cross the human transmission barrier. While these findings do not directly imply that small ruminants TSE agents have caused TSEs in exposed humans, they clearly point out the need to consider this possibility.

## The rising concern of chronic wasting disease

CWD was first recognized in the 1960s, although it was not confirmed to be a prion disease until 1980 [18]. Since its first identification in Colorado, the CWD-affected area has considerably expanded. The disease has now been identified in 21 US states and two Canadian provinces (Saskatchewan and Alberta) and has become endemic in most of these regions (Fig 1E & 1F). However, the discovery of CWD in Saskatchewan deer about 100 kilometers from the Manitoba border has put Manitoba province on alert. The prevalence of CWD in free-ranging cervids varies across North America but can be as high as 30% in some areas [18].

Outside North America, CWD infection has been confirmed in captive cervids in South Korea, as a result of importation of sub-clinically infected animals from Canada [19]. In April 2016, CWD was found in a free-ranging reindeer in Norway and then later (June 2016) in two moose in a different region of Norway [20]. It is unclear at this stage, if CWD presence in Norway has any relationship with the epidemics in North America.

CWD strains and their prevalence remain incompletely characterized. Currently available epidemiological data indicate no clear association between the occurrence of TSEs in humans and exposure to CWD. Transmission experiments in animal models support the view that a substantial species barrier limits the transmissibility of CWD prions to humans. However, in vitro conversion assays have demonstrated that at least certain CWD isolates have the capacity to propagate using human PrP as a substrate [21, 22].

In the USA, hunting product sales and activities exceed $38.3 billion and generate $11.8 billion in state and federal tax revenues annually. The cultural importance of gaming and the weight of hunting industry strongly balance the uncertainties about the zoonotic potential of CWD prions and at this stage, no policy has been implemented at the federal level to mitigate human exposure risks to cervids TSEs agents. However, CDC and the Canadian food inspection agency provided warnings and precautions to hunters and venison consumers about the potential risks associated with CWD.

## What future for TSE risk-control measures?

The active TSE epidemic-surveillance programs, combined with the “total ban”on the use of processed animal proteins in feed and the SRM policy enforced since 2001 by all the EU member states have demonstrated their strong efficacy in controlling animal TSEs and limiting human exposure to animal TSE agents.

These measures clearly go beyond the international standards established by the OIE (emphasizing minimization of the risk of foodborne diseases and control measures considered in general farm and animal health management, i.e., “from farm to fork”) for TSE control and eradication. They have both a direct and indirect (loss of competitiveness) economical cost for countries that implemented them.

Today, the phasing out of BSE epidemics, combined with the renewal of decision makers in the EU administration and the increasing pressure of industry, lead the EU commission to consider the discontinuation of current measures and to align the EU TSE legislation with OIE standards. The first step in this direction was the revision of the cattle SRM policy in 2015. The next step in the EU commission agenda is the abrogation of the SRM measures for small ruminant that was already informally presented to EU member states. On the other side of the Atlantic Ocean, health authorities are aligned to the hypothesis that CWD did not represent a threat for human health.

Many uncertainties related to the zoonotic potential of animal prions, recent discoveries (like atypical BSE and atypical scrapie), and the proportion of chronic wasting disease epidemics raise doubts about these choices, and decades will be needed to determine if these decisions were wise… or not.
